# Myelin water imaging from accelerated 3D-GRASE acquisitions using subspace constrained reconstruction

**DOI:** 10.1007/s10334-025-01276-w

**Published:** 2025-07-18

**Authors:** Riwaj Byanju, Stefan Klein, Alexandra Cristobal-Huerta, Juan A. Hernandez-Tamames, Dirk H. J. Poot

**Affiliations:** Department of Radiology and Nuclear Medicine, Erasmus MC, Rotterdam, The Netherlands

**Keywords:** Myelin water imaging, Myelin water imaging, Brain, Accelerated scan, Undersample, Subspace constrained reconstruction, GRASE

## Abstract

**Purpose:**

Quantitative MRI markers, such as myelin water fraction (MWF) and geometric mean $T_2$ (IET2) (the intra-/extra-cellular water compartment), can be biomarkers for various brain disorders. However, these markers require acquiring multi-echo spin-echo images which requires long scan times. Undersampled 3D-GRAdient Echo and Spin Echo (3D-GRASE) scans with parallel imaging have been used for faster scans. Still, further acceleration is desirable. Reconstruction techniques that utilize redundancy along the echoes could be employed to achieve artifact-free maps at higher acceleration. This work examines the possibility of using one such technique, subspace constrained reconstruction (SCR), for further accelerating the 3D-GRASE scan.

**Methods:**

We propose two techniques to undersample the 3D-GRASE acquisition and exploit the redundancy across echoes. We retrospectively undersample fully sampled data from phantom and in-vivo acquisition to test these techniques. We compared our results for mapping MWF and IET2 to a reference multi-spin-echo technique. Additionally, we compare the proposed, state-of-the-art, and reference techniques with prospectively undersampled in-vivo acquisitions.

**Results:**

The RMSD of the MWF in retrospectively undersampled data was worse for the proposed techniques than the state-of-the-art. However, for IET2, RMSD was similar or slightly improved. In prospectively undersampled scans, undersampling artifacts deteriorated MWF maps, but not IET2 maps, which were within 10 ms of the reference map.

**Conclusion:**

Our findings suggest that exploiting redundancy across echoes does not result in additional acceleration beyond the current state-of-the-art for MWF mapping, while it is possible to accelerate beyond state-of-the-art for IET2 mapping.

## Introduction

Myelin water imaging (MWI), the study of myelin content through magnetic resonance imaging (MRI), is of interest as it is non-invasive and can lead to the early diagnosis of diseases, such as Multiple Sclerosis [1–8]. One of the imaging biomarkers used is myelin water fraction (MWF), which is defined as the ratio of water trapped between the myelin bilayers to the total water present. The water trapped between the myelin bilayers has a shorter transverse relaxation time ($T_2 \le 40$ ms) than the water outside. Therefore, MWF can be measured by probing the influence of multiple $T_2$ times present in the signal from each voxel [9]. Histological studies have validated its use as a biomarker for myelin content in tissues [10–13]. Another biomarker from myelin water imaging is the geometric mean $T_2$ of the intra- and extra-cellular water peak ( $\textrm{IET}_{2}$) [14]. Both MWF and $\textrm{IET}_{2}$ may have clinical relevance [15].

The multiple spin-echo (MSE) sequence is considered a reference method for obtaining MWI and has been shown to be reproducible across sites and hardware [16]. However, its clinical application is limited due to its long scan time, which could be 15–20 min for a few slices [7]. The 3D gradient and spin-echo (3D-GRASE) sequence allows additional gradient recalled echoes within the same repetition time (TR) and hence enables acquiring more information in the same scan time than MSE [17]. Prasloski et al. [7] showed that using 3D-GRASE and parallel imaging scans could be accelerated and whole-brain mapping with voxel size of $1 \times 1 \times 5$ mm would be possible within 15 min. Piredda et al. [18] further accelerated the 3D-GRASE scan using parallel imaging with the CAIPIRINHA undersampling pattern and a 64-channel coil [19] to obtain an isotropic map of 1.6 mm resolution in approximately 10 min. However, further reduction in scan time is desirable for clinical application, especially since such high-SNR 64-channel coils are not widely available.

It has recently been shown that using advanced reconstruction techniques that exploit redundancy across echoes, multi-echo acquisitions such as 3D-GRASE can be accelerated further [20–24]. Usually, such approaches use an echo-dependent undersampling pattern where different k-space positions are sampled in each echo. Application of these techniques to 3D-GRASE could enable faster scans for MWF mapping. The reconstruction techniques in these approaches can be divided into two categories as suggested by Zhao et al. [21], though the boundary is not always sharp:

1. The first category of techniques directly estimates parameter maps from undersampled k-space data skipping the image reconstruction. Such techniques formulate the estimation as a non-linear inverse problem, with the forward model taking the desired tissue parameters as input and predicting undersampled k-space data [21, 23, 24]. The relationship between the desired tissue parameters and the k-space is usually non-linear and thus requires a non-linear optimization routine for parameter estimation. The implementation of such an approach for MWF is challenging. MWF requires the model to consider each voxel’s signal to have multiple $T_2$ decays, unlike conventional relaxometry where mono-exponential decay is sufficient. Even when estimated from the reconstructed magnitude echo images, estimation of the contribution of each $T_2$ is poorly conditioned and requires constraints that force the contribution of each component to be non-negative [9]. Such constraints are applied voxel-wise considering the signal decay across the echoes. The implementation of such constraints while fitting in the k-space domain where each sample influences all voxels is not straightforward.
2. The second category of techniques simultaneously reconstructs all echo images before conventional parameter estimation by exploiting the redundancy across the echoes [25–30], but without explicit parameter estimation. Application of such techniques for MWI has been proposed for the MSE Carr Purcell Meiboom Gill sequence [31, 32] and variable flip angle echo planar time-resolved imaging [33]. Reconstruction of the echo images allows conventional MWF mapping on magnitude echo images using the non-negative least-squares (NNLS) estimation [9].

Subspace constrained reconstruction (SCR) falls in the second category of the techniques described above, exploiting the low-rank nature of data across the echoes [25–30, 34]. The central assumption behind SCR is that a linear subspace exists that describes the signal evolution across echoes sufficiently to extract the underlying tissue parameters. Usually, the subspace is derived from a set of possible signal evolutions called the dictionary. The dictionary can be generated from analytical models considering the distribution of tissue parameters and acquisition settings [34–36] or from the acquired data itself [25]. The dictionary is decomposed into factor matrices using the singular value decomposition (SVD). A number of components that have the most energy are chosen from the resulting matrix.

Conventionally, the extra gradient recalled echoes (GE) in the 3D-GRASE alongside a spin-echo (SE) are used to sample the same k-space as the SE. This arrangement naturally provides an acceleration factor proportional to the extra GEs. For instance, two GEs alongside each SE would accelerate the scan by a factor 3. However, previous studies have shown that such configurations of 3D-GRASE could lead to imaging artifacts due to the difference between GE and SE [37]. The difference is due to the $T_{2}^{'}$ effect and B0-inhomogeneities present in the scan. A B0-compensation step is required to minimize the effect of the B0-inhomogeneities. Conventionally, in such approaches, the phase across the frequency-encoding dimension is recorded before applying phase-encoding gradients, often known as zero-phase encodes. These are used to remove the phase variations caused by the B0-inhomogeneities in the frequency-encoding dimension. In the context of MWF mapping, Piredda et al. [18] used this approach with two GE alongside each SE to gain a factor 3 acceleration and parallel imaging for additional acceleration with a factor 2–6.

This work explores two novel ways to accelerate 3D-GRASE scans by exploiting the redundancy across the echoes. In the first approach, we propose undersampling the 3D-GRASE scan similarly to Piredda et al. [18]; however, we use an echo-dependent undersampling pattern and perform SCR. In the second approach, we propose to separate k-spaces for SE and GE and undersample with an echo-dependent undersampling pattern. The SCR performed on such acquisition allows accounting for SE and GE differences, potentially removing the blurring artifacts observed when GE and SE are placed into the same k-space. We also propose a B0-inhomogeneity compensation step for the second approach. We analyze various aspects of SCR needed for the reconstruction, such as the choice of dictionary and the number of components used to define the subspace. We evaluate the MWF and $\textrm{IET}_{2}$ maps reconstructed from both approaches by comparing them to the reference MSE scan and the state-of-the-art technique proposed by Piredda et al. [18].

## Methods

### Conventional MWF and $\textrm{IET}_{2}$ mapping

Typically, MWF and $\textrm{IET}_{2}$ are computed from the magnitude of echo images reconstructed from a MSE scan. Let ${\varvec{S}}^{\text {MSE}}_{ \varvec{x}} \in {\mathbb {C}}^{J}$ represent the transversal magnetization in the voxel indexed by $\varvec{x}$, a multi-dimensional index for spatial domain $\Omega _{x}$, and *J* echoes. The magnitude of signal from voxels with multi-exponential $T_2$ decay represented by $|{\varvec{S}}^{\text {MSE}}_{\varvec{x}}|$ can be modeled as

1 $$\begin{aligned} |{\varvec{S}}^{\text {MSE}}_{\varvec{x}}| = \sum\limits_{t=1}^{n} {s}_{t,\varvec{x}} \text {EPG}(\varvec{\mathcal {E}}, {T_{2,t}}), \end{aligned}$$

where EPG is an Extended Phase Graph simulation of the magnetization of all *J* echoes [38], *n* is the number of $T_2$ times spaced logarithmically, indexed by $t = [1,n]$, $s_{t, \varvec{x}} \ge 0$ is the unknown (non-negative) contribution of relaxation time $T_{2,t}$ at voxel *x*, $\varvec{\mathcal {E}}$ is the set of echo times [14], and |.| is returning element-wise absolute values. Estimating $s_{t, \varvec{x}}$ from $|\varvec{S}^{\text {MSE}}_{\varvec{x}}|$ requires application of NNLS [9]. Conventionally, $\varvec{\mathcal {E}} = [10, 20, 30, \ldots , 320]$ ms and $T_2$ ranges from 15 ms to 2000 ms with increments of 13.4%, such that $n = 40$ [9, 39]. Then, the MWF and $\textrm{IET}_{2}$ are computed using

2 $$\begin{aligned} \text {MWF} _{\varvec{x}} = \frac{ \sum \nolimits _{t=1}^{8} s_{t, \varvec{x}} }{ \sum \nolimits _{t = 1}^{n} s_{t, \varvec{x}} } \end{aligned}$$

and

3 $$\begin{aligned} { \textrm{IET}_{2}}_{\varvec{x}} = \exp {\left( \frac{ \sum \nolimits _{t=9}^{21} s_{t, \varvec{x}} \ln (T_{2, t})}{ \sum \nolimits _{t=9}^{21} s_{t, \varvec{x}} } \right) }, \end{aligned}$$

where $t = 8$, $t = 9$ and $t = 21$ are equivalent to $T_2 = 36$ ms, $T_2 = 40.9$ ms, and $T_2 = 184.4$ ms, respectively [40].

### Subspace constrained reconstruction

If $\Omega _{k}$ is the k-space domain indexed by multi-dimensional index $\varvec{k}$, ||.|| represents number of elements in the domain, the matrix $\varvec{\mu }_c \in \mathbb {C}^{||\Omega _{k}|| \times J}$ contains predictions of the measurements performed with one of the coils out of *C* indexed by $c= [1, C]$ and is defined as

4 $$\begin{aligned} \varvec{\mu }_{c} = \varvec{U} \odot \varvec{\mathcal {F}} \varvec{\mathcal {C}_{c}} \varvec{S}, \end{aligned}$$

where $\varvec{U} \in \{0, 1\}^{||\Omega _k|| \times J}$ is the binary matrix representing an undersampling mask, $\odot$ represents element-wise multiplication, $\mathcal {F}$ is the Fourier operator, $\mathcal {C}_{c} \in \mathbb {C}^{||\Omega _x|| \times ||\Omega _x||}$ is a diagonal matrix with with the sensitivity for channel *c*, and $\varvec{S} \in \mathbb {C}^{||\Omega _x|| \times J}$ denotes the echo images. The principle behind the SCR is the low-rank nature of the echo images $\varvec{S}$, which allows them to be approximated as

5 $$\begin{aligned} \varvec{S} = \varvec{\alpha } \varvec{\Phi }, \end{aligned}$$

where $\varvec{\Phi }\in \mathbb {C}^{d \times J}$ is called a subspace basis function and $\varvec{\alpha } \in \mathbb {C}^{||\Omega _x|| \times d}$ are the coefficients of the subspace where $d < J$ represents the number of subspace components used. $\varvec{\Phi }$ primarily should be designed, such that the difference $\varvec{S} - \varvec{\alpha } \varvec{\Phi }$ is small for all realistic $\varvec{S}$. From Eqs. 4 and 5, $\varvec{\mu }_{c}$ can be written as

6 $$\begin{aligned} \varvec{\mu }^{SCR}_{c} = \varvec{U} \odot \varvec{\mathcal {F}} \varvec{\mathcal {C}_{c}} \varvec{\alpha } \varvec{\Phi }. \end{aligned}$$

The reconstruction of $\varvec{S}$ can be formulated as a maximum-likelihood estimation to recover $\varvec{\alpha }$ from noisy measured k-space data from each coil $\varvec{Z}_{c} \in \mathbb {C}^{||\Omega _{k}|| \times J}$. The noise in the measured k-space can be assumed to be normally distributed with independent real and imaginary channel. If the variance of noise is $\sigma ^2$, the estimation problem can be defined by

7 $$\begin{aligned} \hat{\varvec{\alpha }} = \arg \max _{\varvec{\alpha }} L(\varvec{\alpha }) = \arg \min _{\varvec{\alpha }} \frac{1}{2\sigma } \sum _{1}^{C} || \varvec{Z}_{c} - \varvec{\mu }_{c}^{SCR} ||_2^2. \end{aligned}$$

After estimation of $\hat{\varvec{\alpha }}$, echo images $\varvec{S}$ can be computed using Eq. 5. Note that by defining $\varvec{S}$ in terms of $\varvec{\alpha } \varvec{\Phi }$, the number of unknowns decreased from *J* per voxel to *d* per voxel. The magnitude of $\varvec{S}$ can then be used for estimating $s_{t,\varvec{x}}$ for each voxel using the NNLS algorithm proposed by Mackay et al. [9] and further developed to enable *B*1 inhomogeneity correction by Prasloski et al. [39].

#### Dictionaries

Various dictionaries $\varvec{D}$ have been proposed to derive $\varvec{\Phi }$ by taking SVD of $\varvec{D}$. Some are data driven [25, 41] and others use a model of signal evolution across echoes [28]. Moreover, different values of *d* have been used for each application [28]. We describe the selection of $\varvec{D}$ and *d* in the following subsections.

##### Model-based dictionary ($\varvec{D}^{\text {Model}}$)

An analytical model predicts the possible signal evolution for the expected distribution of tissue parameters. For MWF mapping, the multi-component relaxation model defined in Eq. 2 can be used to create dictionary $\varvec{D}^{\text {Model}}$. The change in signal $\varvec{S}_{ \varvec{x}}$ due to each component can be computed by taking the partial derivative with respect to each of them as shown by Eq. 8 [42]. For a given range of $T_2$ and the acquisition setting $\varvec{\mathcal {E}}$, a dictionary $\varvec{D}^{\text {Model}}$ can be produced

8 $$\begin{aligned} \begin{array}{lll} {\varvec{D}}^{\text {Model}}_{t} = \frac{ \partial {S}^{\text {MSE}}_{ \varvec{x}} }{ \partial s_{t,\varvec{x}} }. \end{array} \end{aligned}$$

***Dictionary from fully sampled patch*** ($\varvec{D}^{\text {Patch}}$) Petzscher et al. [25] proposed to acquire ‘training data’ in the same scan to build a dictionary for $T_1$ and $T_2$ mapping. A fully sampled patch in the center of k-space acquired for each echo to produce low-resolution echo images. These low-resolution images can be used as the dictionary $\varvec{D}^{\text {Patch}}$.

#### Selection of dictionaries and number of subspace components

As metric to select the best result among the different dictionaries and number of subspace components (*d*), we used root-mean-square difference (RMSD) with respect to the reference images or maps (which one will be clear from context)

9 $$\begin{aligned} \textrm{RMSD} = \sqrt{\tfrac{1}{{|\Omega ^{ROI}_{x}|}}{\sum _{\varvec{x} \in \Omega ^{ROI}_{x}} \left( {R}_{\varvec{x}} - {A}_{\varvec{x}}\right) ^2}}, \end{aligned}$$

where $\varvec{R} \in \mathbb {R}^{\Omega _{x}}$ and $\varvec{A} \in \mathbb {R}^{\Omega _{x}}$ represent maps or images taken from the region of interest $\Omega ^{ROI}_{x} \subset \Omega _{x}$.

**Fig. 1:**
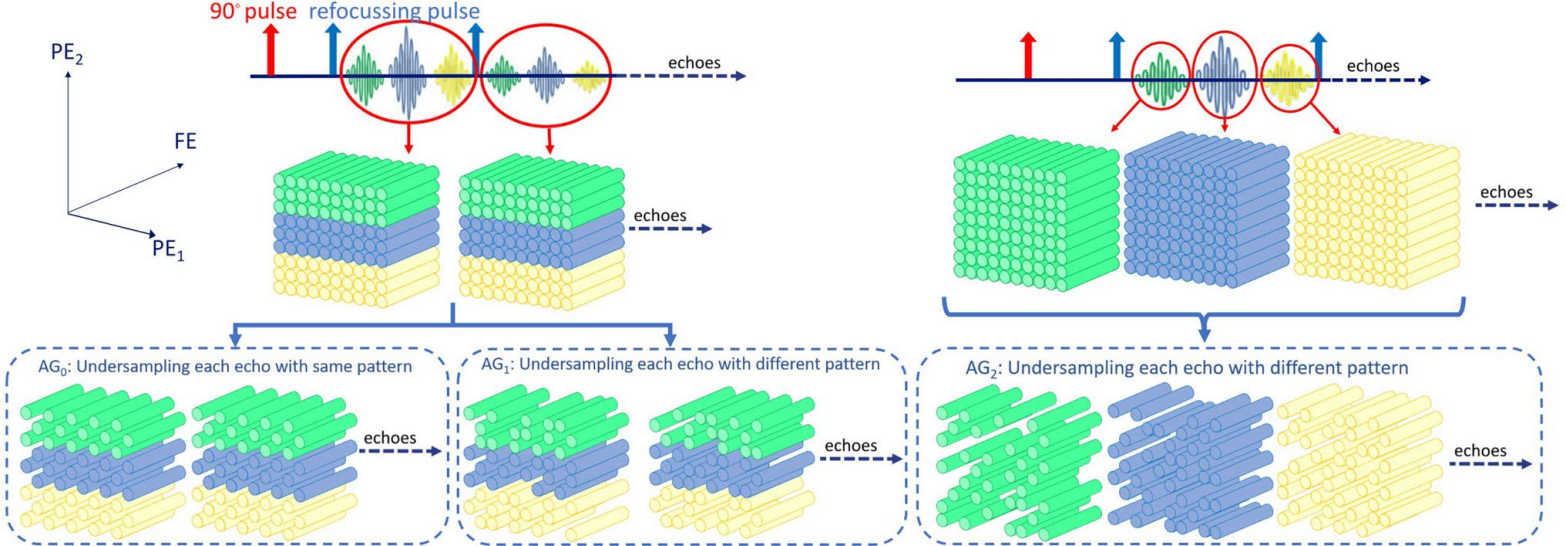
The two proposed strategies for undersampling 3D-GRASE scans with two GE around each SE are used in this work. The cylinders show frequency-encoding lines. The green color represents the first GE, blue represents SE, and yellow represents the second gradient echo

### Accelerating 3D-GRASE sequence

This section starts with a brief description of the state-of-the-art accelerated 3D-GRASE scan proposed by Piredda et al. [18]. Unlike their approach, we intend to exploit redundancy across echoes using SCR additionally to the parallel imaging-based redundancy. Therefore, in the subsequent subsections, we propose two ways to configure the 3D-GRASE for undersampled acquisitions, such that it is more suited for joint reconstruction of echo images.

#### Accelerated 3D-GRASE state-of-the-art ($\textrm{AG}_0$)

Piredda et al. [18] used parallel imaging to reconstruct images from an undersampled 3D-GRASE scan. As illustrated in Fig. 1, they used the conventional configuration of 3D-GRASE, where two GEs were used along with the SE to sample the same k-space. However, they undersampled the scan further using the CAIPIRINHA undersampling pattern [43]. They used the same undersampling pattern across the echoes. We refer to such approaches where SE and GEs are recorded in the same k-space and the same undersampling pattern is used in each echo as $\textrm{AG}_0$. The B0-inhomogeneity correction was performed using the zero-phase encodes [17, 18].

#### Accelerated 3D-GRASE 1 ($\textrm{AG}_1$)

As shown in Fig. 1, for this acceleration strategy, we propose to use a distribution of GE and SE similar to $\textrm{AG}_0$; however, we sample different points in k-space for each echo. We refer to such approach as $\textrm{AG}_1$. Using a different undersampling pattern for each echo would promote information sharing across the echoes, which is suitable for joint reconstruction.

We used a low-discrepancy pseudo-random sampling pattern generated using a Halton sequence that was shown to be time-efficient for 3D-GRASE scans [44] referred here after as $\textrm{AG}^\textrm{Halton}_{1}$. As additional comparison, we also use CAIPIRINHA undersampling pattern [43], which is shifted along one of the spatial dimensions by one position along the echoes referred to as $\textrm{AG}^\textrm{CAIPIs}_{1}$. Similar to $\textrm{AG}_0$, the B0-inhomogeneity correction was performed using the zero-phase encodes.

#### Accelerated 3D-GRASE 2 ($\textrm{AG}_2$)

As shown in Fig. 1, in the $\textrm{AG}_2$ configuration, we use GE and SE to fill different k-spaces. Without undersampling, this would require three times the amount of data to fill all k-spaces compared to the MSE scan in the same scan time. However, we propose to undersample each k-space, so that the overall scan time is the same or shorter than the conventional 3D-GRASE scans. We use the Halton sequence and shifted CAIPIRINHA for generating an undersampling pattern similar to $\textrm{AG}_1$ and refer to it as $\textrm{AG}^\textrm{Halton}_2$ and $\textrm{AG}^\textrm{CAIPIs}_2$, respectively.

The $\textrm{AG}_2$ configuration requires the model to consider the difference between GE and SE for dictionary generation. The signal from a single voxel in such cases can be modeled as [14, 45]

10 $$\begin{aligned} {S}^{GRASE}_{\varvec{x}, j} = {S}^{MSE}_{\varvec{x}, j} e^{- |\Delta _{j}|{{R{2}^{'}}_{\varvec{x}}} + i{\Delta {B0}}_{\varvec{x}}{\Delta _{j} } }, \end{aligned}$$

where $\Delta _{j}$ is the time offset of echo $j = [1,J]$ from the spin echo, ${{R2}^{'}}_{\varvec{x}}$ is the component of ${R{2}}^{^*}_{\varvec{x}}$ accounting for the de-phasing of magnetisation and $\Delta {B0}_{\varvec{x}}$ is the local offset in the main magnetic field. Note that in this case, the number of echoes $J^{\textrm{AG}_2} = nSE \times (nGE+1)$ where *nGE* is number of gradient echoes present with each spin echo.

To construct the model-based dictionary for $\textrm{AG}_{2}$, a similar approach as in Eq. 8 is taken where additionally, realistic values of $\Delta {B0}_{\varvec{x}}$ and ${{R2}^{'}}_{\varvec{x}}$ are taken

11 $$\begin{aligned} \begin{array}{lll} {D}^{\text {Model}, \textrm{AG}_2}_{j,t} = \frac{ \partial S^{GRASE}_{j,\varvec{x}}}{ \partial s_{t,\varvec{x}} } =&{D}^{\text {Model}}_{j,t}e^{- |{\Delta _{j}|{{R2}^{'}_{\varvec{x}}} + i{\Delta {B0}_{\varvec{x}}}\Delta _{j} }}. \end{array} \end{aligned}$$

Note that we now rename the model-based dictionary defined by Eq. 8 as ${\varvec{D}}^{\text {Model}}$ to distinguish the dictionaries between $\textrm{AG}_1$ and $\textrm{AG}_2$. We use extended phase graph simulations to compute ${\varvec{D}}^{\text {Model}}$ [39]. To add $B_1$ variations to the dictionary, we introduce flip angles (FA) variations in the range from $120^\circ$ to $180^\circ$.

***B0-inhomogeneity correction*** A realistic range of *B*0 values in the dictionary generation step can lead to non-compact subspaces (large *d*). We hypothesize that by compensating the majority of the *B*0 effect, the dictionary can be more compact again. Specifically, we propose a B0-inhomogeneity compensation step for $\textrm{AG}_2$ similar to the approach proposed by Liu et al. [46]. A patch of fully sampled region ($12 \times 12$) is acquired from the center of the k-space for each echo similar to those required for constructing ${\varvec{D}}^{\text {Patch}}$. We use the patch of fully sampled region to generate low-resolution images $\varvec{S^{\text {Patch}}}$. The phase changes observed for voxel $\varvec{x}$ across the echoes $j-1$ and *j*, i.e. $\angle {S^{\text {Patch}}_{\varvec{x}, j-1}} - \angle {S^{\text {Patch}}_{\varvec{x}, j}}$, were used to compensate for the effect of B0-inhomogeneities by decreasing the phase changes across the GE and SE images during reconstruction. An experiment to verify how much of overall ${\Delta B0}_{\varvec{x}}$ can be computed using the patch of k-space and the resulting compression in the corresponding subspace is provided in the appendix A.

### Experiments

We performed experiments to investigate the accuracy of images, MWF, and $\textrm{IET}_{2}$ maps from SCR images using retrospectively and prospectively undersampled 3D-GRASE acquisitions. Both phantom and in-vivo experiments are performed as described in the following subsections.

#### Phantom data

We acquired a fully sampled scan of the ISMRM model 130 phantom [47]. The acquisition was performed with a 3.0 T clinical scanner (Discovery MR750, GE Healthcare, Waukesha, WI) with a 32-channel head coil. The acquisition settings are similar to Piredda et al. [18] and given in Table 1. The repetition time (TR) was set to 1800 ms instead of 1000 ms [18] due to a SAR limit of the scanner. The total scan time was 5 h and 23 min. To accommodate the phase correction technique of $\textrm{AG}_0$ and $\textrm{AG}_1$, a single echo train with zero-phase encode amplitude was acquired prior to the echo trains with readout.

**Table 1:** Acquisition settings used in the phantom and volunteer scans. Note that the integer inside the round brackets for acceleration factor denotes part of the overall acceleration achieved by sampling k-space with SE and GE. Dimensions: *FE* = frequency encode, $PE_1$ = phase encoding 1, and $PE_2$ = phase encoding 2

Scans	Phantom scan	Volunteer scans				
		Reference	$\textrm{AG}_0$	$\textrm{AG}_1$	$\textrm{AG}_2$	$T_1$-weighted 3D FSPGR
Acquisition matrix $FE \times PE_1 \times PE_2$	$128 \times 128 \times 84$	$128 \times 128 \times 7$	$128 \times 128 \times 56$	$128 \times 128 \times 56$	$128 \times 128 \times 56$	$256 \times 256 \times 184$
Orientation	LR: FE AP: $PE_1$ SI: $PE_2$	LR: $PE_1$ AP: *FE* SI: $PE_2$	LR: $PE_1$ AP: *FE* SI: $PE_2$	LR: $PE_1$ AP: *FE* SI: $PE_2$	LR: $PE_1$ AP: *FE* SI: $PE_2$	LR: $PE_1$ AP: *FE* SI: $PE_2$
Resolution (mm)						
$FE \times PE_1 \times PE_2$	$1.6 \times 1.6 \times 1.6$	$2.4 \times 2.4 \times 2.4$	$2.4 \times 2.4 \times 2.4$	$2.4 \times 2.4 \times 2.4$	$2.4 \times 2.4 \times 2.4$	$1 \times 1 \times 1.4$
Number of coils	32	32	32	32	32	32
Acceleration factor	1	1	9 (3)	9 (3)	9	1
Scan time without fully sampled patch	5 h 23 min	14 min	8 min	8 min	8 min	6 min
Scan time for the fully sampled patch	NA	NA	3 min	3 min	3 min	NA
Repetition time (TR) (ms)	1800	1100	1100	1100	1100	6068
Echo train length	32	32	32	32	32	NA
Additional GE along side SE	2	2	2	2	2	NA
Echo spacing (ms)	10.4	10.2	10.2	10.2	10.2	NA
Total echoes	96	96	32	32	96	NA
Refocussing flip angles	$180^\circ$	$180^\circ$	$180^\circ$	$180^\circ$	$180^\circ$	NA
Delay between SE and GE (ms)	1	2.5	2.5	2.5	2.5	NA

***Reference SE*** Reference images were constructed from each of the fully sampled SE echoes. For each echo, a complex valued image was reconstructed by FFT and coil combination with coil sensitivity maps obtained via the ESPIRiT technique [48] by BART [49] on the first SE.

#### Experiment 1

In this experiment, we compare retrospectively undersampled $\textrm{AG}^\textrm{Halton}_{2}$ and $\textrm{AG}_{0}$, with an overall acceleration factor of 3 ($R=3$). The purpose is to observe if any artifacts occur in $\textrm{AG}_{0}$ due to the mixing of SE and GE in k-space and if $\textrm{AG}_{2}$ produces results similar to the reference SE. $\textrm{AG}_{0}$ configuration with $R = 3$ represents a conventional 3D-GRASE scan where a fully sampled k-space is formed using two GEs around each SE to fill a k-space. We used the same reconstruction technique as the reference SE for $\textrm{AG}_{0}$. For $\textrm{AG}_{2}$, we reconstructed the echo images using SCR with $\varvec{D}^{\text {Model}, \textrm{AG}_{2}}$, which was created with the scan settings listed in Table 1. This includes 40 logarithmically spaced $T_{2}$ values between 15 ms and 2000 ms, 30 logarithmically spaced $T^{'}_{2}$ values between 10 ms and 1000 ms, and 200 entries of ${\Delta B0}_{\varvec{x}}$ between (-150, 150) Hz. We visually compared the resulting images, specifically looking for artifacts related to SE and GE mixing in $\textrm{AG}_{1}$ and any artifacts due to undersampling in $\textrm{AG}_{2}$ configuration.

#### Experiment 2

This experiment evaluates the quality of the echo images for three configurations $\textrm{AG}_0$, $\textrm{AG}^\textrm{Halton}_1$ and, $\textrm{AG}^\textrm{Halton}_{2}$ and four accelerations factors $R \in \{ 3$, 6, 12, $18 \}$. The configurations were retrospectively undersampled from the fully sampled reference scan. A fully sampled region of $12 \times 12$ was left in the center of the k-space in each echo in the undersampling patterns. The reconstructed images are shown, and the RMSD computed using the magnitude images is used to evaluate the quality with respect to the reference SE images.

The reconstruction for $\textrm{AG}_0$ configuration was performed for each echo separately using ESPIRiT reconstruction technique with the BART toolbox [48, 49]. Note that this differs from the original work, where online reconstruction was performed in the scanner using GRAPPA [18].

The $\textrm{AG}^\textrm{Halton}_{1}$ and $\textrm{AG}^\textrm{Halton}_{2}$ configurations were reconstructed using SCR based on $\varvec{D}^{\text {Model}, \textrm{AG}_{1}}$ and $\varvec{D}^{\text {Model}, \textrm{AG}_{2}}$. The $\varvec{D}^{\text {Model}}$ was the same as described previously for the case of $R = 3$ except for the range of ${\Delta B0}_{\varvec{x}}$, which was between $(-20, 20)$ Hz.

#### In-vivo data

#### Fully sampled data

We conducted two fully sampled scans acquiring the complete set of GE and SE on two healthy volunteers (1 and 2) using the same scanner as phantom experiment with settings shown in Table 1 for retrospective undersampling experiments. These acquisitions focused on seven central brain slices to limit scan time. Additionally, a $T_1$ weighted image was acquired using a 3D FSPGR sequence (Table 1).

***Reference data*** Conventional inverse Fourier transform-based reconstruction was employed (Sect. 2.4.1), and SEs from this experiment served as a reference. We applied the MWF mapping procedure using the same NNLS fitting settings as Piredda et al. [18] with FA ranging from $120^\circ$ to $180^\circ$ in $1^\circ$ increments, obtaining MWF and $\textrm{IET}_{2}$ maps.

#### Experiment 3

This experiment evaluates the quality of the MWF and $\textrm{IET}_{2}$ maps for three configurations $\textrm{AG}_0$, $\textrm{AG}^\textrm{Halton}_1$, and $\textrm{AG}^\textrm{Halton}_2$ and six accelerations factors $R \in \{ 3$, 6, 9, 12, 15, $18 \}$ by retrospectively undersampling the fully sampled data. We selected the central slice (no. 4) from the reference scan. We treated Anterior–Posterior and Left–Right as $PE_1$ and $PE_2$ for retrospective undersampling experiments, generating $\textrm{AG}_0$, $\textrm{AG}^\textrm{Halton}_{1}$, $\textrm{AG}^\textrm{CAIPIs}_{1}$, $\textrm{AG}^\textrm{Halton}_2$, and $\textrm{AG}^\textrm{CAIPIs}_2$ with overall accelerations of $R \in \{3, 6, 9, 12, 15, 18\}$ from both the volunteer scans. For computing the coil maps and the $\varvec{D}^{\text {Patch}}$ in case of $\textrm{AG}_1$, a $12 \times 12$ patch in the center of k-space in each echo was left fully sampled.

The retrospectively undersampled data for volunteer 1 were reconstructed using all possible dictionary and subspace component combinations. We reconstructed the SE and GE images from one volunteer scan using the same process as the reference SE data from the phantom experiment described in Sect. 2.4.1.

The $T_1$ weighted images were processed by Freesurfer [50] to create a segmentation mask. The $T_1$-weighted images were registered to the echo image 14 of the slices of reference scan using FSL toolbox [51]. The white matter mask from the segmentation was transformed into the space of the reference scan. The masks were used as ROI to compute RMSD between accelerated maps and reference maps. The dictionary and the number of subspaces with the lowest RMSD were used to reconstruct the volunteer 2 data for each configuration, undersampling pattern, and acceleration factor.

#### Experiment 4

We tested the possibility of prospective undersampling. We performed a prospectively undersampled scan on a single healthy volunteer (3) using three different configurations: $\textrm{AG}_0$, $\textrm{AG}^\textrm{Halton}_1$, and $\textrm{AG}^\textrm{Halton}_2$. The overall acceleration factor was $R = 9$ including a fully sampled 12 x12 center patch. Excluding that patch the acceleration factor was $R = 12$. Further acquisition settings are given in Table 1. Additionally, we obtained a fully sampled scan with seven slices from the central region of the brain for comparison. We aligned the central seven slices of the accelerated and reference scans based on their world coordinates during the scan. The dictionary and number of subspace components were selected based on the results of Experiment 3. A $T_1$ weighted image was acquired and used for tissue segmentation as in Experiment 3 and registration to image $t = 14$ (E = 140 ms) of the $\textrm{AG}_0$ scan. The white matter mask from the segmentation was warped with the registration result to form a region of interest in which the RMSD between reference and accelerated scans is evaluated.

## Results

The used implementation of the SCR took about 1 min to reconstruct a single frequency-encoding line on an 8-core Intel Core i7 PC with 16 GB of RAM, with only small variations for different *d* and acceleration factors.

### Phantom experiments

#### Experiment 1

**Fig. 2:**
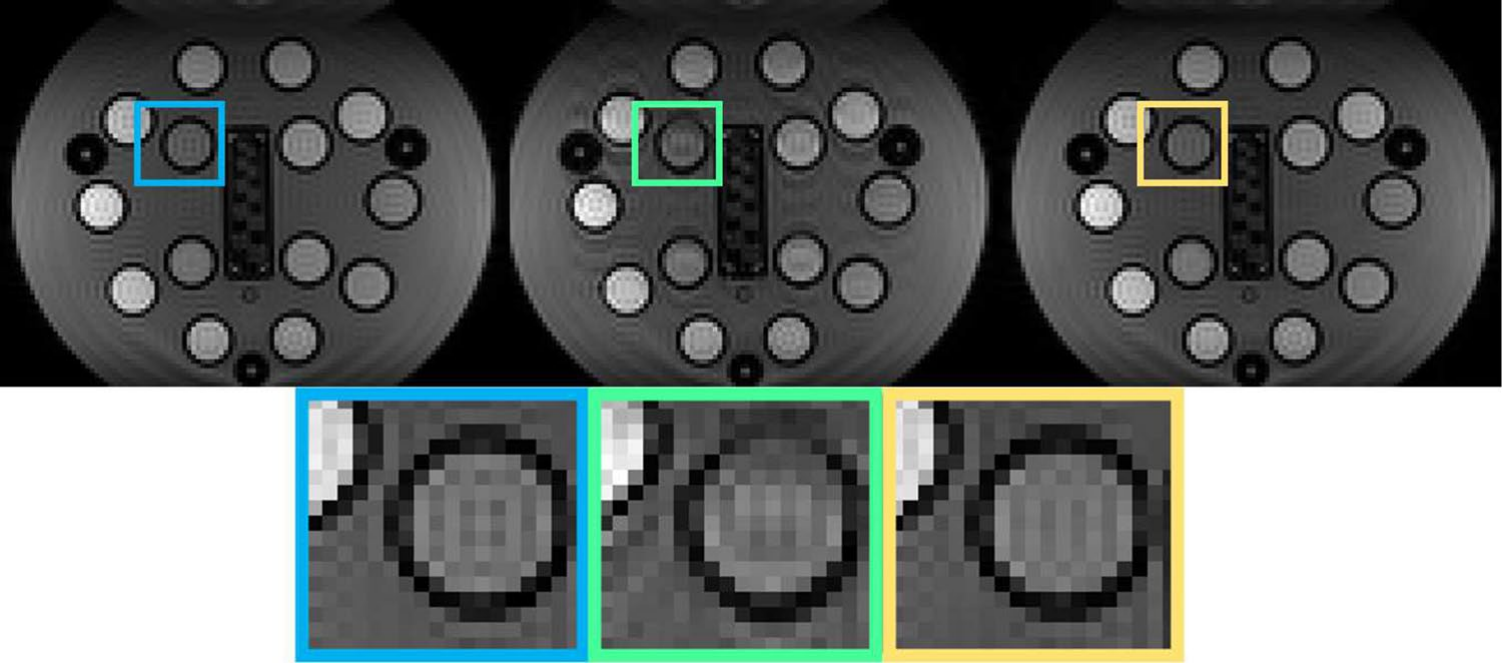
Comparison of $\textrm{AG}_0$ and $\textrm{AG}_2$ configuration with R = 3. First row: the first SE reconstructed from fully sampled data (left), $\textrm{AG}_0$ configuration with R = 3 (center) and $\textrm{AG}_2$ configuration with R = 3 (right). Second row: zoom-in on the colored boxes. Note that horizontal direction shows frequency encoding dimension and vertical dimension is the phase-encoding dimension 2

**Fig. 3:**
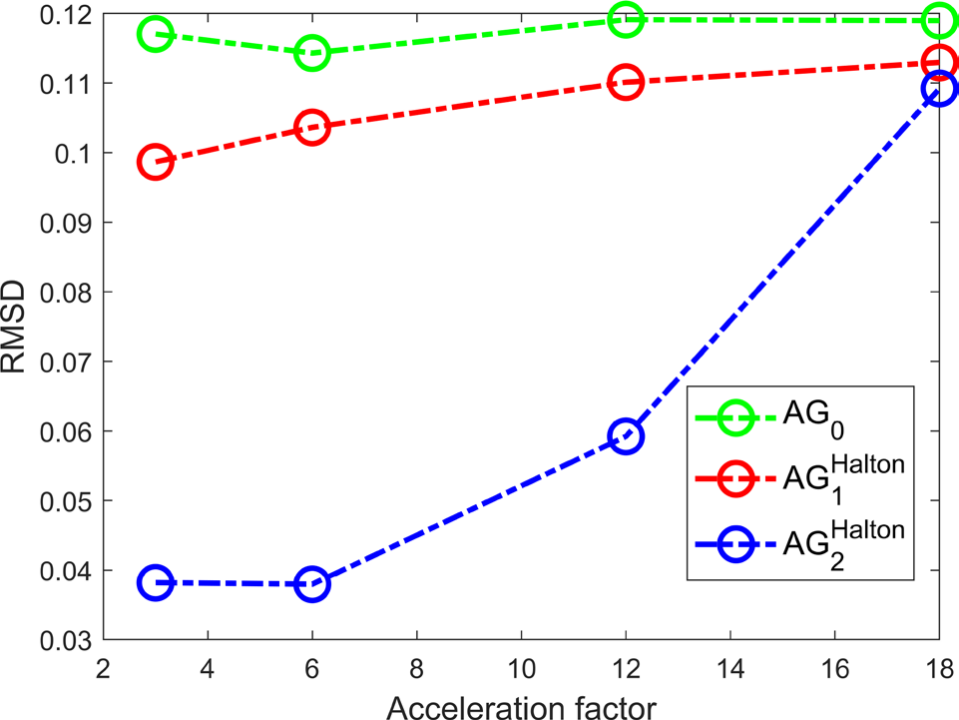
RMSD computed for spin-echo images for $\textrm{AG}_0$, $\textrm{AG}_1$, and $\textrm{AG}_2$ shown for $R = 3, 6, 12, 18$ computed by comparing with SE reference images

Figure 2 shows the first SE images from the $\textrm{AG}_0$ and $\textrm{AG}_2$ retrospectively undersampled to $R = 3$ along with the reference SE image. Note that for $\textrm{AG}_0$, this is a full k-space. As shown in the zoomed-in part, the blurriness observed along the phase encoding direction in $\textrm{AG}_0$ is absent in the result from $\textrm{AG}_2$ and SE. The reconstructed images of $\textrm{AG}_2$ have an RMSD three times lower than $\textrm{AG}_0$, as depicted in the graph in Fig. 3. Moreover, based on the residual in the reconstruction of $\textrm{AG}_2$, the model-based reconstructed images with a subspace component of 12 matched up to $99.22\%$ of the measured data.

#### Experiment 2

**Fig. 4:**
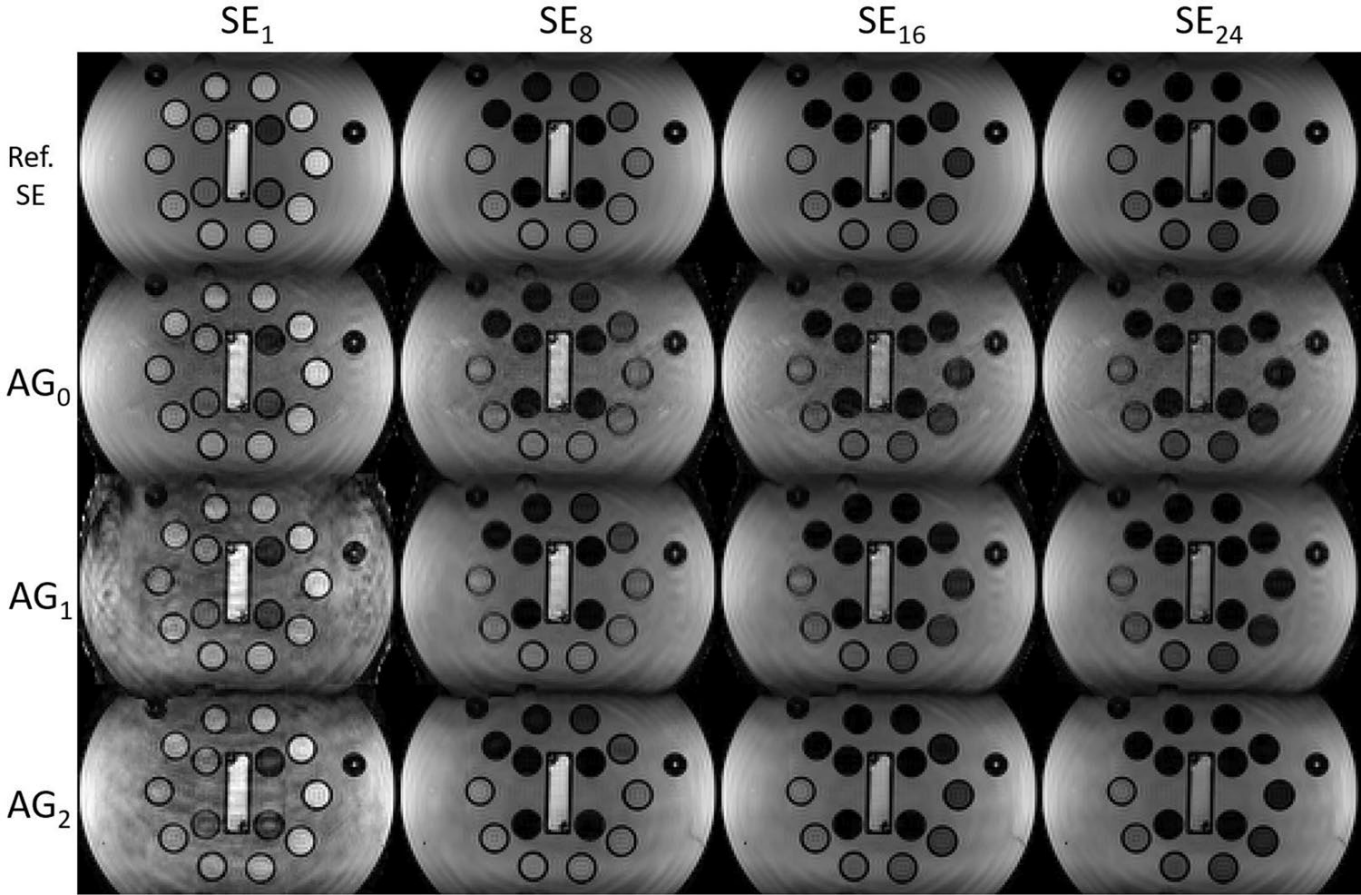
Echo images for $\textrm{AG}_0$, $\textrm{AG}_1$ and $\textrm{AG}_2$ shown for $R = 18$ for spin echoes 1, 8, 16, and 24. The vertical direction is phase-encoding direction 2, while horizontal direction is frequency-encoding direction

Figure 4 shows the echo images from $\textrm{AG}_{0}$, $\textrm{AG}_{1}$, and $\textrm{AG}_2$ for $R = 18$ with the SE reference images. Note that reconstructed images from $\textrm{AG}_{0}$ have similar artifacts in each echo, while for $\textrm{AG}_1$ and $\textrm{AG}_2$, the first echo has increased artifacts and the other echoes have less artifacts. Observe that images from $\textrm{AG}_1$ have stronger artifacts than $\textrm{AG}_2$, particularly in the first spin echo.

Figure 3 shows that $\textrm{AG}_2^{Halton}$ has the lowest RMSD with respect to the reference images.

### In-vivo experiment

#### Experiment 3

**Fig. 5:**
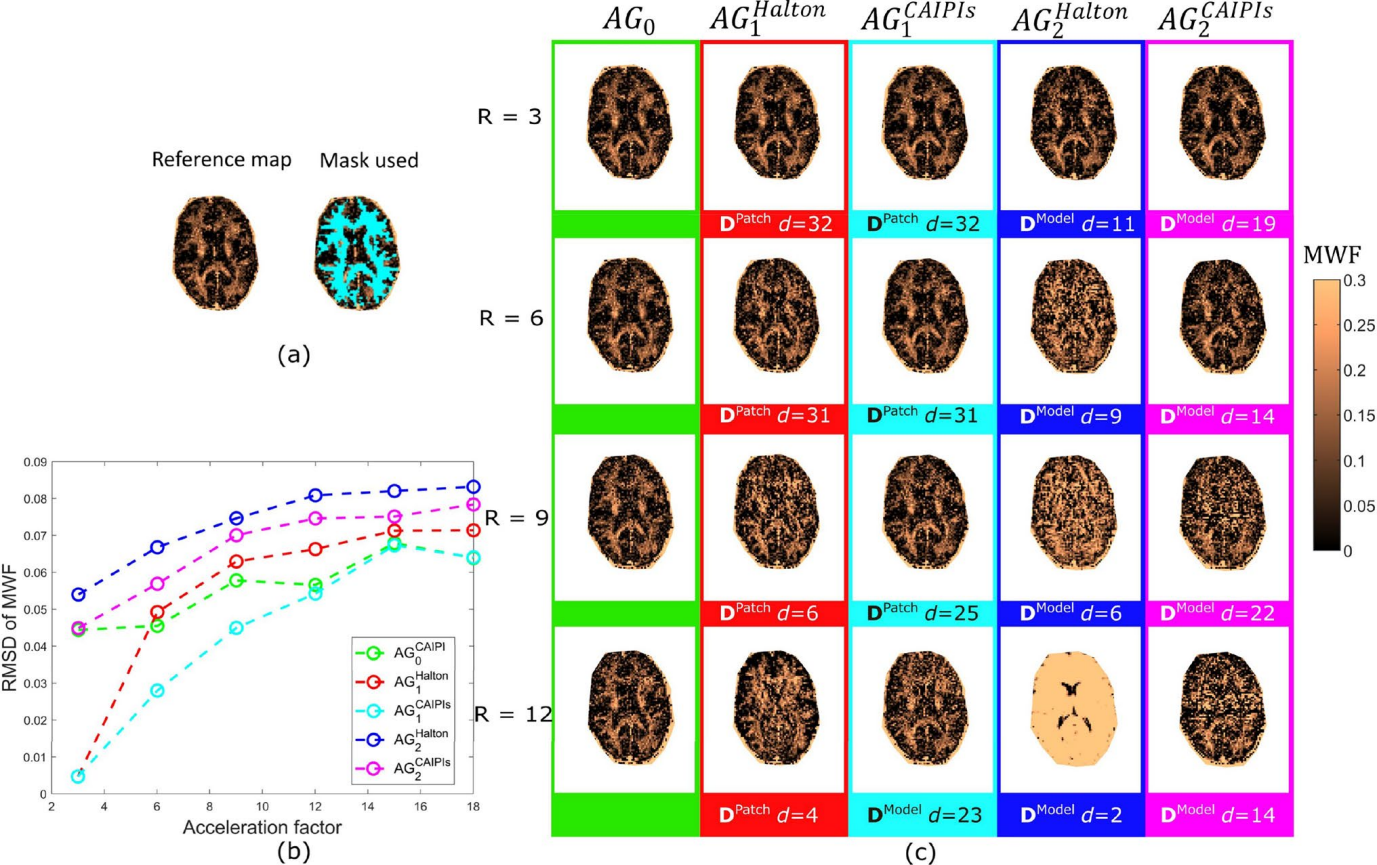
Retrospective undersampling results from volunteer 1. **a** Reference MWF map reconstructed using fully sampled SE. **b** Plot showing the RMSD computed for MWF maps across different acceleration factors for different configuration and undersampling strategies. **c** The MWF maps reconstructed using different strategies and undersampling patterns. The maps with lowest RMSD with respect to the reference map have been shown

**Fig. 6:**
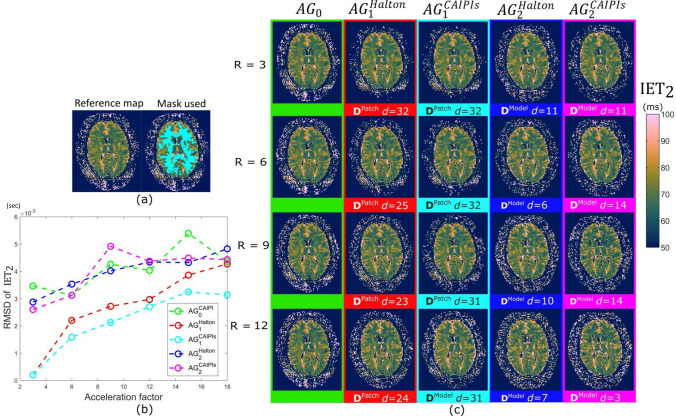
Retrospective undersampling results from volunteer 1. **a** Reference $\textrm{IET}_{2}$ map reconstructed using fully sampled SE. **b** Plot showing the RMSD computed for $\textrm{IET}_{2}$ maps across different acceleration factors for different configuration and undersampling strategies. **c** The $\textrm{IET}_{2}$ maps reconstructed using different strategies and undersampling patterns. The maps with lowest RMSD with respect to the reference map have been shown

The results of the first volunteer are shown in Figs. 5 and 6, while those of the second volunteer are shown in Appendix B. Figure 5 shows the MWF maps for the different undersampling patterns, and acceleration factors. Similarly, Fig. 6 shows the same from $\textrm{IET}_{2}$ maps. The dictionary $D^{\text {Patch}}$ produced the best MWF and $\textrm{IET}_{2}$ maps on the $AG_1$ configuration, while the number of subspace components generally decreased with with increasing acceleration factor.

Among all the acceleration factors, for MWF $\textrm{AG}^\textrm{CAIPIs}_1$ displays the lowest RMSD. For acceleration factor 3, $\textrm{AG}^\textrm{Halton}_1$ is better than $AG_0$, and for higher factors, the difference between $\textrm{AG}^\textrm{Halton}_1$ and $\textrm{AG}^\textrm{CAIPIs}_0$ is smaller than that between $\textrm{AG}^\textrm{CAIPIs}_0$ and $AG^\textrm{CAIPIs}_1$. Figure 5c illustrates that the $\textrm{AG}_2$ approach with both undersampling patterns displays artifacts in the MWF maps, which is in line with the RMSD values shown in 5b for these patterns.

In the $\textrm{IET}_{2}$ maps, the strategies using SCR with mixed echoes, i.e., $\textrm{AG}_1$, showed better results across different acceleration factors than $\textrm{AG}_2$, with $AG^\textrm{CAIPIs}_1$ marginally showing the best results. Visually, the $\textrm{IET}_{2}$ maps suffer less from the artifacts than MWF maps, even for higher acceleration factors.

#### Experiment 4

**Fig. 7:**
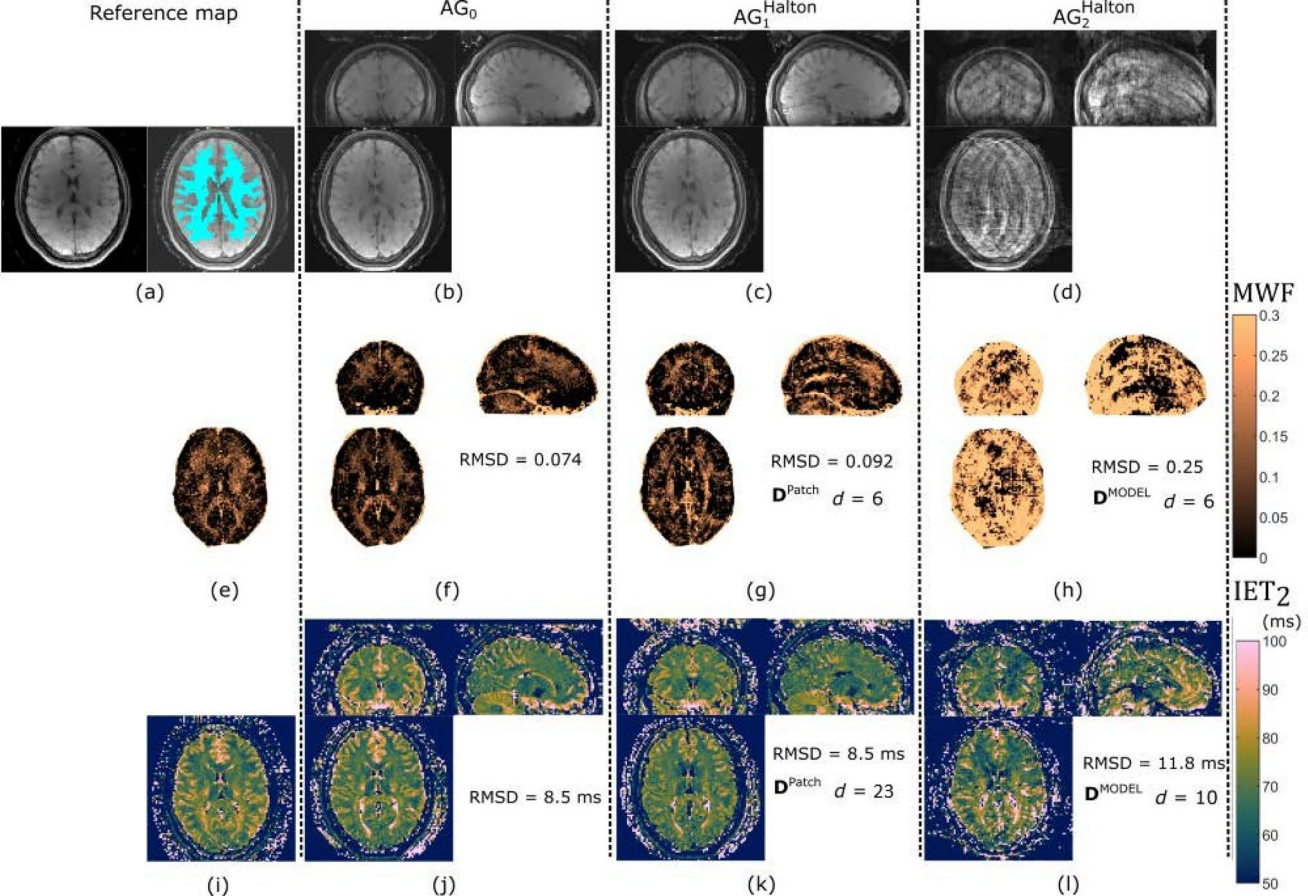
Prospective undersampling results in volunteer 1. **a** Magnitude of first SE image from reference scan with the turquoise mask showing the ROI (left) and without the mask (right). Magnitude image of first SE from **b** $\textrm{AG}_0$, **c** $\textrm{AG}^\textrm{Halton}_1$ and **d** $\textrm{AG}^\textrm{Halton}_2$ scan. Note that the intensity scale of each image is not equal. MWF maps from **e** reference scan (central slice), **f** $\textrm{AG}_0$, **g** $\textrm{AG}^\textrm{Halton}_1$, and **h** $\textrm{AG}^\textrm{Halton}_2$. ${IET}_{2}$ maps derived from **i** reference scan (central slice), **j** $\textrm{AG}_0$, **k** $\textrm{AG}^\textrm{Halton}_1$, and **l** $\textrm{AG}^\textrm{Halton}_2$

Figure 7 displays the reconstructed images and maps in the prospective undersampling experiment as well as the reconstruction settings used and the RMSD with respect to the maps from the reference scan. In Fig. 7 a, note the artifacts in the first echo of $\textrm{AG}^\textrm{Halton}_2$ which is absent from the reference image as well as $\textrm{AG}_0$ image shown in Fig. 7 b. For the MWF maps, the lowest RMSD was obtained with $\textrm{AG}_0$, which visually shows less noise than the reference map and fewer artifacts than $\textrm{AG}^\textrm{Halton}_1$ and $AG^\textrm{Halton}_2$. For the $\textrm{IET}_{2}$, the RMSD differed only by $10\%$, with $\textrm{AG}_0$ and $\textrm{AG}^\textrm{Halton}_1$ scoring equal. Therefore, both in terms of MWF and $\textrm{IET}_{2}$ $\textrm{AG}_0$ scored best in the white matter ROI with respect to the reference scan.

## Discussion

This work aimed at accelerating the 3D-GRASE scan for MWI by exploiting the redundancy across the echoes. Two configurations to undersample the 3D-GRASE acquisition were explored, each with two undersampling patterns. We used SCR to consider redundancy across echoes in the reconstruction. We performed phantom and in-vivo scans to investigate whether reconstructed maps and images are comparable to those of reference scans and the state-of-the-art $\textrm{AG}_0$. Retrospective undersampling experiments on phantom data showed promising results by producing fewer artifacts than the $\textrm{AG}_0$ configuration. However, the in-vivo experiments showed increased artifacts for MWF and $\textrm{IET}_{2}$ with high acceleration factors.

We maintained identical spin-echo echo times between fully sampled and prospectively undersampled scans to ensure that any differences in the images result solely from variations in sampling. In the AG1 approach, while we do combine different echo times in k-space, this involves only spin and gradient echoes, not multiple spin echoes.

Both in the phantom and in-vivo acquisitions, the first echo image of the SCR reconstructed image series showed artifacts. This has a strong effect on the MWF mapping as the apparent proportion of spins with $0 \le T_2 \le 40$ ms is strongly dependent on the shortest echo time images. This means that artifacts in the first echo image propagates to the MWF maps. We observed that reducing the number of subspace components *d* in the SCR resulted in MWF maps that were closer to the reference maps in terms of RMSD. However, reduced *d* were not sufficient for mapping MWF maps in such cases. Hence appearing as constant values across the voxels. The artifacts in the reconstructed images show that there needs to be more redundancy to overcome the effects of undersampling. Previous work based on simulation [42] suggested that redundancy across the echoes was sufficient for artifact-free and accurate MWF mapping. However, it was only in the case of acquisition with a sufficient level of SNR, that is, SNR $\ge$ 40 [42]. Therefore, it could be that the information sharing across echoes is possible only if certain level of SNR is available.

In the phantom experiment with an overall acceleration factor of 3, the blurring observed in the phase-encoding dimension 2 in $\textrm{AG}_0$ is absent in the $\textrm{AG}_2$ approach. The main reason for this improvement in AG2 is that in this method, the entire 3D phase (and magnitude) differences between the GE and SE are considered during the SCR. In $\textrm{AG}_0$ and $\textrm{AG}_1$, the ‘zero phase encode’ reference echo train only allows compensating phase differences in the frequency-encoding dimension. While there is a clear improvement in the phantom, this effect is not clear in the in-vivo experiments.

A limitation of the B0-inhomogeneity correction method is that it relies on the assumption that the B0 inhomogeneity varies smoothly, i.e., only small $\Delta B0_{x}$ are left after subtracting the smooth map reconstructed from the fully sampled center of k-space region. This assumption may not hold if there are local iron deposits in the brain as is the case in some diseases [52].

The poor performance of all the configurations for MWF mapping beyond an acceleration factor of 9 could be due to increased artifacts in the first echo of the reconstructed images. When evaluating for each echo the fraction of noise that can be explained by the subspace, we observe that it is largely independently estimated for the range of *d* values that is selected and, hence, the later echoes do not (sufficiently) help to reduce undersampling artifacts in the first echo. The artifacts in the first echo are noticeable in the phantom experiments. We also tried excluding the first echo in the MWF evaluation; however, this did not improve the results. The $\textrm{IET}_{2}$ maps, which are much less influenced by the first echo image, show much better performance for SCR reconstructed techniques, particularly for $\textrm{AG}_1$, than $\textrm{AG}_0$ approaches. The $\textrm{IET}_{2}$ maps reconstructed from the prospective in-vivo scans exhibited values greater than 5 ms, while the normal variation of $\textrm{IET}_{2}$ with age has been reported to be a mean of approximately 4 ms and a standard deviation of about 5 ms [53]. This suggests that the $\textrm{IET}_{2}$ maps reconstructed from the prospective in-vivo scans are not sufficiently sensitive to be useful for clinical applications. By performing SCR both with a data driven dictionary as well as a model-based dictionary, the poor performance can not be only attributed to a model misfit or noise in the dictionary.

In our model, we did not incorporate the effects of microstructure-induced frequency shifts within the different water compartments, as observed in the previous studies [54–56]. While such shifts have been observed in other contexts, they are expected to have a negligible impact on the gradient echo data we analyze due to the small echo time differences $\Delta _j$.

Using only the redundancy across echoes was not enough to accelerate further than state-of-the-art. Other prior information such as those learned through deep learning approaches could be another way to accelerate further [57, 58].

The $\textrm{AG}_1^\textrm{CAIPIs}$ configuration yielded the best results in the in-vivo experiment, achieving the lowest RMSD up to an acceleration factor of 12. For acceleration factor of 3, where the $\textrm{AG}_0$ and $\textrm{AG}_1$ have the same k-space data, the redundancy across the echoes in this approach acts as a denoising step, similar to the principle component analysis used in MWF mapping.

The $\textrm{AG}_1^\textrm{Halton}$ approach performed worse in comparison to $\textrm{AG}^\textrm{CAIPIs}_1$, likely due to the lower parallel imaging performance of the low-discrepancy Halton sequence-based patterns compared to the CAIPIRINHA patterns [43]. However, their performances were similar for acceleration factors greater than 15, indicating a loss of parallel-imaging-based advantage with higher undersampling. The shifting performed on the CAIPIs pattern preserves its low-discrepancy property and capitalizes on the advantages of the CAIPIRINHA pattern, potentially explaining why this approach produced the best results for MWF and $\textrm{IET}_{2}$ maps. The $\textrm{AG}_0$ approach from Piredda et al. [18] demonstrated that good MWF maps could be obtained with an acceleration factor of up to 12. We obtained similar maps using retrospective undersampling at a lower resolution than what was proposed in their study. The reason could be the differences in the acquisition setup, particularly the receiver coils. They used a 64-channel coil, which is known to produce lower g-factor penalties at high acceleration factors compared to the 32-channel coils used in our study. [19].

## Conclusion

We explored the possibility of accelerating 3D-GRASE scans for faster MWF and $\textrm{IET}_{2}$ mapping by exploiting the redundancy across the echoes. The accelerated scans did not show evidence that further acceleration of the scans beyond the state-of-the-art for MWF mapping is feasible with SCR. Possible reasons for the poor performance of proposed accelerated scans for MWF mapping could be insufficient redundancy between information in the first echo and the rest of the echoes. One of the proposed acceleration techniques produced images free of blurring artifacts usually observed in such scans and has the potential for use in contrast-weighted structural imaging.

## Figures and Tables

**Fig. 8:**
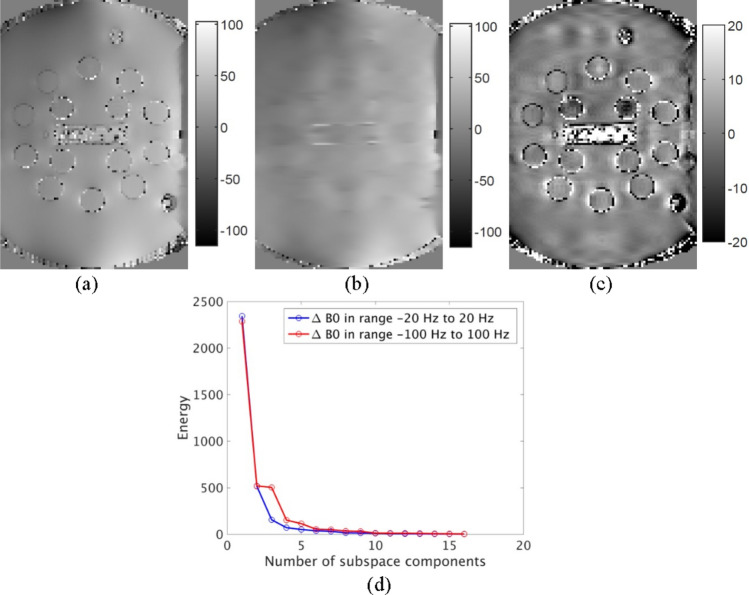
**a** ${\Delta {B0}}_{\varvec{x}}$ from fully sampled data in rad/s, **b** ${\Delta {B0}}_{\varvec{x}}$ from fully sampled patch in rad/s. **c** Difference map. **d** Singular values of basis function obtained after SVD ranges of ${\Delta {B0}}_{\varvec{x}}$ with *B*0 compensation (red) and without *B*0 compensation (blue)

**Fig. 9:**
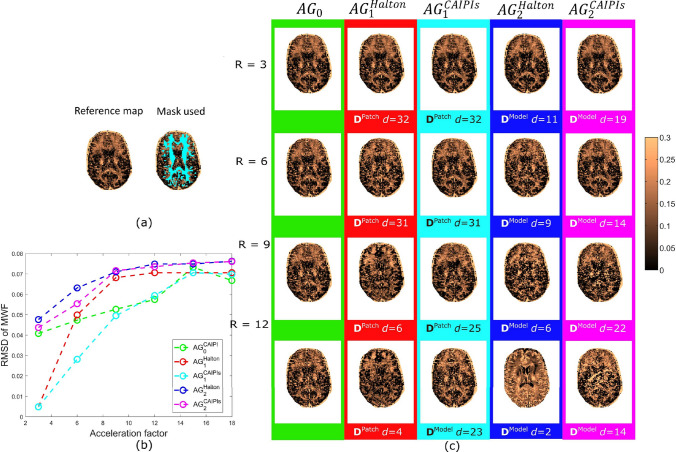
Retrospective undersampling results from volunteer 2. **a** Reference MWF map reconstructed using fully sampled SE. **b** Plot showing the RMSD computed for MWF maps across different acceleration factors for different configuration and undersampling strategies. **c** The MWF maps reconstructed using different strategies and undersampling patterns. The maps with lowest RMSD with respect to the reference map have been shown

**Fig. 10:**
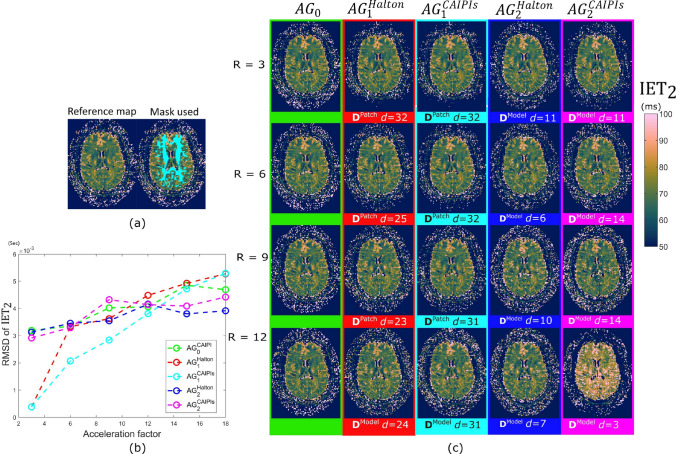
Retrospective undersampling results from volunteer 2. **a** Reference ${IET}_{2}$ map reconstructed using fully sampled SE. **b** Plot showing the RMSD computed for ${IET}_{2}$ maps across different acceleration factors for different configuration and undersampling strategies. **c** The ${IET}_{2}$ maps reconstructed using different strategies and undersampling patterns. The maps with lowest RMSD with respect to the reference map have been shown

## Data Availability

The data that support the findings of this study are not openly available due to reasons of sensitivity and are available from the corresponding author upon reasonable request. Data are located in controlled access data storage at Erasmus MC.

## Appendix A: Subspace compression in 3D-GRASE through $B_0$ inhomogeneity compensation

In this experiment, we examine if the signal evolution in a $\textrm{AG}_2$ configuration of 3D-GRASE can be compressed to subspace with lower *d* after a ${{\Delta } B0}_{\varvec{x}}$ compensation step based on a patch of k-space from the center. We compute the ${{\Delta }{B0}}_{\varvec{x}}$ using the fully sampled region in the center as well as the entire k-space (fully sampled) and estimate the remaining ${{\Delta }{B0}}_{\varvec{x}}$. Using first two successive gradient echoes ($j = 1$ and $j = 3$), the ${\Delta }{B0}_{\varvec{x}}$ can be computed using Eq. A1. Note that the imaginary part of the logarithm of the ratio of signals is computing the phase difference without wrap-around

A1 $$\begin{aligned} \Delta {B0}_{\varvec{x}} = \tfrac{1}{-2\Delta _{j} i} \mathcal {I} \left\{ \log \left( \frac{S^\textrm{GRASE}_{1,\varvec{x}}}{S^\textrm{GRASE}_{3,\varvec{x}}}\right) \right\} , \end{aligned}$$

where $\mathcal {I}$ is a function to get the imaginary part and $S^\textrm{GRASE}_{1,\varvec{x}}$ is complex valued.

Figure 8 (a and b) shows the ${\Delta B0}_{\varvec{x}}$ map observed in the phantom scan computed from the fully sampled data as well as computed using the central patch of $12 \times 12$ used in *B*0 inhomogeneity correction in $\textrm{AG}_{2}$. The difference map in 8 (c) indicates the difference in ${\Delta B0}_{\varvec{x}}$ is within $(-20, 20)$ Hz except for some areas of the phantom. Figure 8 (d) shows the energy of components of $\varvec{\Phi }$ computed from $\varvec{D}^{\textrm{MODEL}, \textrm{AG}_{2}}$ using the range of ${\Delta B0}_{\varvec{x}}$ in the range$(-100, 100)$ and $(-20, 20)$ Hz.

Using $(-20, 20)$ Hz for generating $\varvec{D}^{\text {Model}, \textrm{AG}_{2}}$ clearly produces more compressed subspace. The difference map in 8 (c) indicates that using the fully sampled patch is sufficient to compensate ${\Delta B0}_{\varvec{x}}$ to the level required for using the lower range of ${\Delta B0}_{\varvec{x}}$ in the dictionary for reconstruction.

## Appendix B: Experiment 3: results from volunteer 2

Figures 9 and 10 show results from the second volunteer. The details are different compared to volunteer 1, especially in the highly accelerated $AG_2$ MWF maps. However, the same patterns and acceleration factors provide (relatively) good quality MWF maps. Hence, these volunteer data support the reasoning and conclusions drawn in the main text.
